# The sodium channel-blocking antiepileptic drug phenytoin inhibits breast tumour growth and metastasis

**DOI:** 10.1186/s12943-014-0277-x

**Published:** 2015-01-27

**Authors:** Michaela Nelson, Ming Yang, Adam A Dowle, Jerry R Thomas, William J Brackenbury

**Affiliations:** Department of Biology, University of York, Heslington, York, YO10 5DD UK

**Keywords:** Antiepileptic, Breast cancer, Metastasis, Phenytoin, Voltage-gated Na^+^ channel

## Abstract

**Background:**

Voltage-gated Na^+^ channels (VGSCs) are heteromeric protein complexes containing pore-forming α subunits and smaller, non-pore-forming β subunits. VGSCs are classically expressed in electrically excitable cells, e.g. neurons. VGSCs are also expressed in tumour cells, including breast cancer (BCa) cells, where they enhance cellular migration and invasion. However, despite extensive work defining in detail the molecular mechanisms underlying the expression of VGSCs and their pro-invasive role in cancer cells, there has been a notable lack of clinically relevant *in vivo* data exploring their value as potential therapeutic targets.

**Findings:**

We have previously reported that the VGSC-blocking antiepileptic drug phenytoin inhibits the migration and invasion of metastatic MDA-MB-231 cells *in vitro*. The purpose of the present study was to establish whether VGSCs might be viable therapeutic targets by testing the effect of phenytoin on tumour growth and metastasis *in vivo*. We found that expression of Na_v_1.5, previously detected in MDA-MB-231 cells *in vitro*, was retained on cells in orthotopic xenografts. Treatment with phenytoin, at a dose equivalent to that used to treat epilepsy (60 mg/kg; daily), significantly reduced tumour growth, without affecting animal weight. Phenytoin also reduced cancer cell proliferation *in vivo* and invasion into surrounding mammary tissue. Finally, phenytoin significantly reduced metastasis to the liver, lungs and spleen.

**Conclusions:**

This is the first study showing that phenytoin reduces breast tumour growth and metastasis *in vivo*. We propose that pharmacologically targeting VGSCs, by repurposing antiepileptic or antiarrhythmic drugs, should be further studied as a potentially novel anti-cancer therapy.

**Electronic supplementary material:**

The online version of this article (doi:10.1186/s12943-014-0277-x) contains supplementary material, which is available to authorized users.

## Findings

Despite recent advances, breast cancer (BCa) is still the leading cause of cancer-related deaths in women [1]. Metastasis, the spread of tumours to secondary sites, is responsible for 90% of these deaths and is rarely curable [2]. Thus, there is an urgent need to identify new molecular targets and curative therapies. Voltage-gated Na^+^ channels (VGSCs) contain a pore-forming α subunit with smaller β subunits. There are nine α subunits, Na_v_1.1-Na_v_1.9, and four β subunits, β1-β4. The β subunits modulate channel function and are cell adhesion molecules (CAMs) [3]. VGSCs transmit electrical activity in cells in the nervous system and regulate neuronal growth and migration during CNS development [4]. VGSCs are clinical targets for a range of disorders, including epilepsy, cardiac arrhythmias, neuropathic pain and depression [5].

VGSCs are widely expressed in traditionally non-excitable cells, including microglia, astrocytes, immune cells, fibroblasts and cancer cells [6]. In the latter, a number of studies have shown that VGSCs contribute to cellular migration and invasion [7]. Na_v_1.5 is up-regulated in breast tumours, associating with recurrence, metastasis, and reduced survival [8,9]. Na_v_1.5 carries a fast inward Na^+^ current in triple negative (lacking estrogen receptor, progesterone receptor and HER2) MDA-MB-231 cells [9-11]. Pharmacological or genetic ablation of this Na^+^ current inhibits *in vitro* cell behaviours associated with the metastatic cascade, including migration, galvanotaxis, and invasion [9-11]. Similar results have been reported in metastatic cell lines from other cancers, suggesting that VGSC expression/activity in cancer may be a general phenomenon [7,12]. Na^+^ current enhances invasion by promoting cysteine cathepsin activity in caveolae *via* allosteric regulation of the Na^+^/H^+^ exchanger type 1 [13], and Na_v_1.5 is a key regulator of a gene network that controls invasion [14]. In addition, the widely used VGSC-blocking Class Ib antiarrhythmic agent and antiepileptic drug (AED) phenytoin (5,5-diphenylhydantoin) reduces the migration and invasion of MDA-MB-231 cells *in vitro* [8]. Furthermore, we have recently shown that the VGSC β1 subunit is also expressed in BCa specimens, and accelerates tumour growth and metastasis in a mouse model [15].

Together, these data highlight the potential for VGSCs as novel molecular targets. However, there remains a paucity of clinically relevant *in vivo* data exploring their potential therapeutic value. The aim of the present study was to study the effect of phenytoin on tumour growth and metastasis in a mouse model of triple negative BCa. We found that systemic phenytoin treatment reduces cellular proliferation, tumour growth, local invasion and metastasis. This is the first *in vivo* study demonstrating the potential therapeutic value of pharmacologically targeting VGSCs in BCa using an AED.

### Phenytoin reduces tumour growth

**Figure 1:**
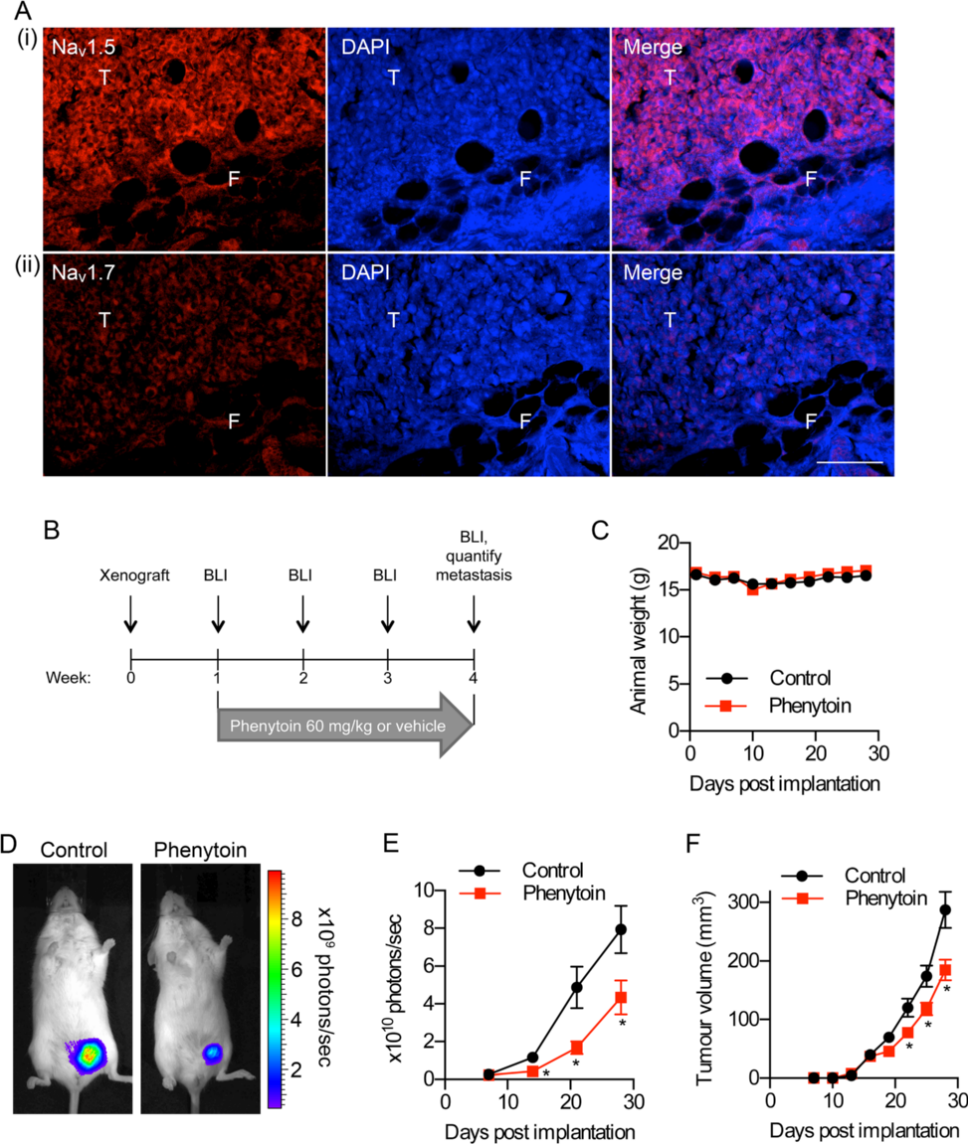
**Effect of phenytoin on breast tumour growth. (A)** Tumour section stained with (i) anti-Na_v_1.5 or (ii) anti-Na_v_1.7 (red) and DAPI (blue). T, tumour, F, fibroadipose tissue. Scale bar, 100 μm. **(B)** Phenytoin dosing protocol. BLI, bioluminescent imaging. **(C)** Weight of mice during the assay. **(D)** Bioluminescent images of control and phenytoin-treated mice, 4 weeks after implantation. **(E)** Bioluminescence measured from primary tumours on the indicated days post-implantation. **(F)** Calculated volume derived from calliper measurement of primary tumours over the same period. Data are mean ± SEM; *P < 0.05 (n = 13 for control, n = 15 for phenytoin).

Na_v_1.5 is expressed on cancer cells from breast tumours in clinical specimens, and in MDA-MB-231 cells cultured *in vitro* [8-11]. Here, we studied VGSC expression in tumours following orthotopic implantation of luciferase-expressing MDA-MB-231 cells into the mammary fat pad of female *Rag2*^*-/-*^*Il2rg*^*-/-*^ mice, a robust model of BCa growth and metastasis [15]. All methods are described in detail in Additional file 1. Na_v_1.5 expression, detected by immunohistochemistry, was retained in the tumours *in vivo* (Figure 1Ai). Na_v_1.7 was also present in the tumours, although expression was weaker (Figure 1Aii). These data agree with previous *in vitro* studies showing that although Na_v_1.5 is the predominant VGSC in MDA-MB-231 cells, accounting for >80% of Na^+^ current, there may be a small contribution from other isotypes, e.g. Na_v_1.7 [9,11]. We have previously shown that phenytoin inhibits Na^+^ current and VGSC-dependent migration in MDA-MB-231 cells in culture, suggesting that pharmacological targeting of VGSCs may have therapeutic utility in BCa [8]. In order to test the effect of phenytoin on BCa progression *in vivo*, we next treated tumour-bearing mice with 60 mg/kg phenytoin or vehicle (by daily intraperitoneal injection) for three weeks, starting one week after orthotopic implantation of MDA-MB-231 cells (Figure 1B). This dosing regimen gave a phenytoin trough level of 9.0 ± 1.0 μg/ml plasma, measured by liquid chromatography-mass spectrometry with single reaction monitoring (LC-SRM-MS) on samples taken 16 h after the last dose, which is within the therapeutic range for epilepsy treatment in rodents (6-23 μg/ml) [16]. We have previously shown that a similar concentration (50 μM) blocks Na^+^ current in MDA-MB-231 cells by 43% [8]. Importantly, there were no obvious signs of toxicity associated with the phenytoin treatment, and animal weight remained constant throughout the experiment (Figure 1C). Photon flux from tumours increased more slowly in phenytoin-treated animals than control-treated animals, indicating that the drug reduced the rate of tumour growth (Figure 1D,E). We also analysed tumour growth by calliper measurement. As with the bioluminescent data, the volume of tumours increased more slowly in phenytoin-treated animals than in control animals, indicating that phenytoin slowed the rate of tumour growth (Figure 1F).

### Phenytoin reduces invasion and proliferation

**Figure 2:**
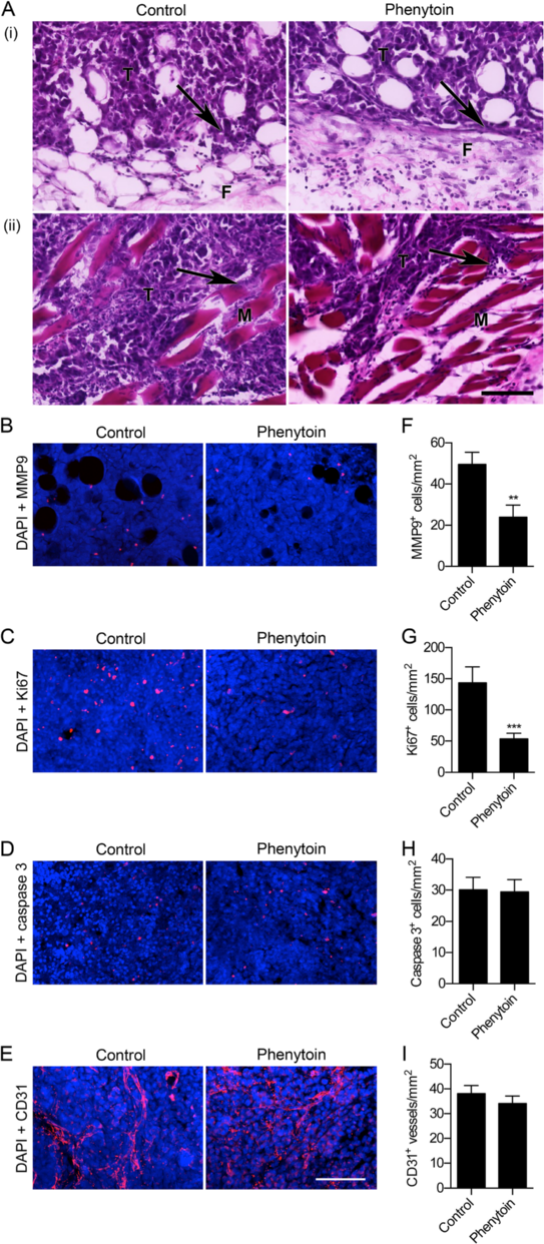
**Effect of phenytoin on invasion, proliferation, apoptosis and angiogenesis. (A)** Tumour sections stained with H&E showing (i) mammary fat pad and (ii) skeletal muscle invasion. Arrows, infiltration of tumour cells (T) into fibroadipose tissue (F) or skeletal muscle fibres (M). **(B)** Tumour stained with anti-MMP9 (red) and DAPI (blue). **(C)** Tumour stained with anti-Ki67 (red) and DAPI (blue). **(D)** Tumour stained with anti-activated caspase-3 (red) and DAPI (blue). **(E)** Blood vessels stained with anti-CD31 (red) and DAPI (blue). **(F)** MMP9^+^ cells/mm^2^ (n = 40) **(G)** Ki67^+^ nuclei/mm^2^ (n = 40). **(H)** Activated caspase-3^+^ cells/mm^2^ (n = 40). **(I)** CD31^+^ blood vessels/mm^2^ (n = 40). Data are mean + SEM; **P < 0.01; ***P < 0.001. Scale bars, 100 μm.

We next studied the effect of phenytoin treatment on the structure and composition of the primary tumours. At the tumour periphery, there was some local invasion into surrounding skeletal muscle and fibroadipose tissue, and this invasion was moderately reduced (indicated by arrows) in phenytoin-treated animals compared to control (Figure 2A). Various MMPs, e.g. MMP9, are expressed in carcinomas, correlating with local invasion [17]. We found that the density of MMP9-expressing cells was significantly reduced by 51.9% in the tumours of phenytoin-treated animals (P < 0.01; Figure 2B,F). Together, these data suggest that phenytoin reduces local invasion from tumours *in vivo*, as it does in the same cells cultured *in vitro* [8].

We found that the prevalence of Ki67-expressing cycling cells was reduced by 62.6% in the tumours of phenytoin-treated animals (P < 0.001; Figure 2C,G). However, the number of apoptotic cells expressing activated caspase-3 was unchanged (Figure 2D,H). Similarly, the phenytoin treatment had no effect on the density of CD31-expressing vascular structures (Figure 2E,I). Together, these data suggest that phenytoin inhibited growth of primary tumours by reducing the number of proliferating cancer cells, rather than by inhibiting angiogenesis or promoting apoptosis. Interestingly, previous studies have indicated that VGSCs do not regulate proliferation of MDA-MB-231 cells in 2D cultures *in vitro* [9,10]. However, the VGSC blocker tetrodotoxin reduces colony growth in 3D Matrigel matrices [18]. Thus, the contribution of VGSCs to tumour growth *in vivo* appears complex, and may be dependent on multiple factors, including heterotypic signalling interactions with adjacent cells or the extracellular matrix [15]. In addition, VGSCs may regulate proliferation *via* reverse Na^+^/Ca^2+^ exchange, as has recently been shown in astrocytes after injury [19].

### Phenytoin reduces metastasis

**Figure 3:**
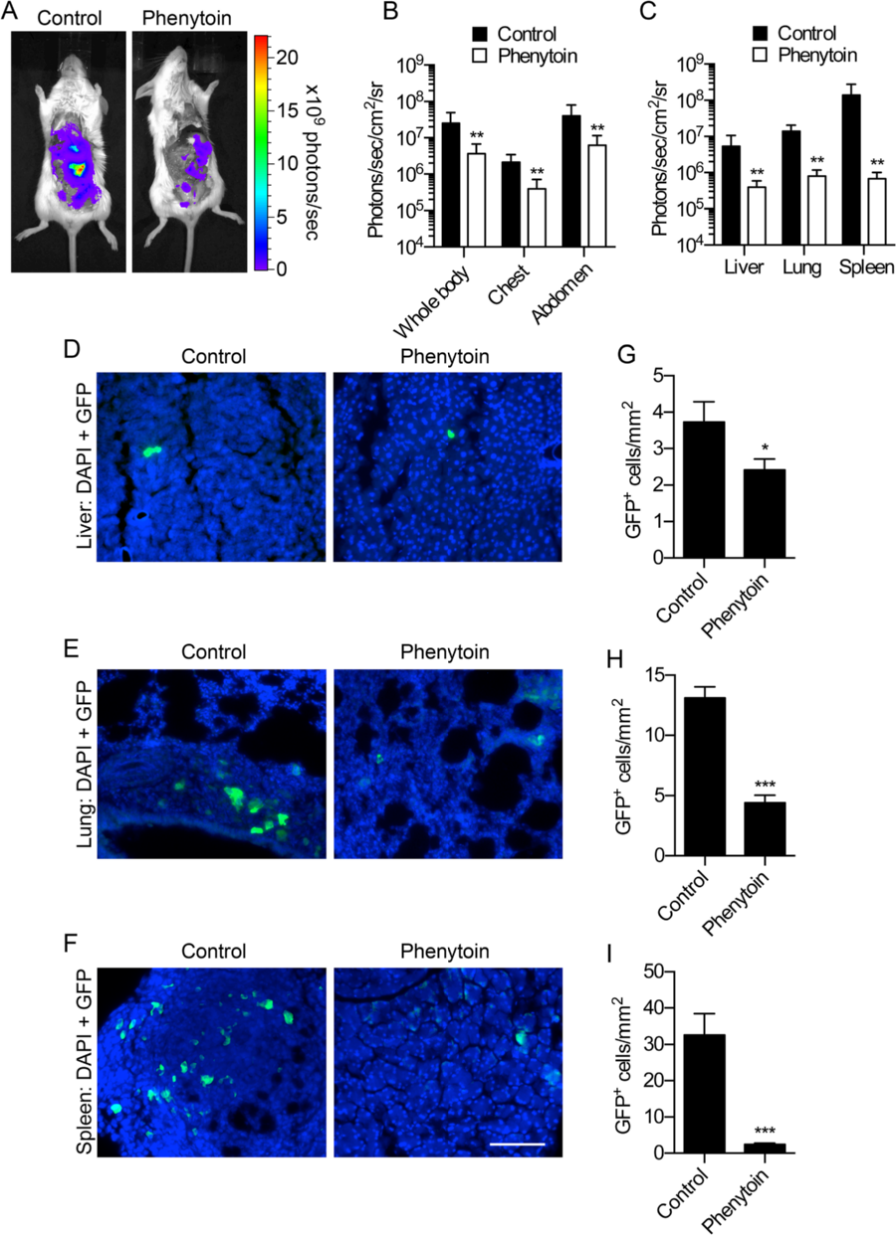
**Effect of phenytoin on metastasis. (A)** Bioluminescent images of metastases in control and phenytoin-treated mice. **(B)** Bioluminescence measured from the indicated anatomical sites (n ≥ 10). **(C)** Bioluminescence measured *ex vivo* from the liver, lungs and spleen (n = 11). Liver **(D)**, lungs **(E)**, and spleen **(F)** stained with anti-GFP (green) and DAPI (blue). **(G,H,I)** GFP^+^ cells/mm^2^ at each site (n ≥ 249). Data are mean + SEM; *P < 0.05; ***P < 0.001. For **(B)** and **(C)**, P < 0.01 between control and phenytoin (two-way ANOVA). Scale bar, 100 μm.

When we monitored metastasis 3 weeks after onset of drug treatment, following *post mortem* resection of the primary tumour (Figure 3A), photon flux was significantly reduced across the whole body, chest and abdomen of phenytoin-treated animals compared to control animals (P < 0.01; Figure 3B). Similarly, there was a notable reduction in photon flux across metastatic sites measured *ex vivo* (P < 0.01; Figure 3C). In order to further study metastasis to these sites at the cellular level, we next measured the number of GFP-expressing tumour cells within tissue sections. We have previously shown that GFP expression is retained in MDA-MB-231 cells at metastatic sites in this mouse model [15]. The number of GFP-expressing cells was moderately reduced in the liver of phenytoin-treated animals by 35.4% (P < 0.05; Figure 3D,G). Phenytoin treatment caused a more robust reduction in the density of metastatic cells in the lungs and spleen, of 66.3% and 92.4%, respectively (P < 0.001; Figure 3E,F,H,I). In summary, phenytoin treatment reduced BCa metastasis *in vivo.*

### Therapeutic potential

We have previously shown that phenytoin inhibits Na^+^ current and significantly reduces migration and invasion of BCa cells *in vitro* [8]. Together with the present data, these findings suggest that targeting VGSC-mediated Na^+^ current with phenytoin may have therapeutic value. Phenytoin also inhibits migration and secretion in prostate cancer cells [20,21], suggesting that it may have broad utility in other cancers. In support of this, tetrodotoxin has been shown to reduce metastasis in a rat prostate cancer model [22]. In the present study, we provide, for the first time, clinically relevant *in vivo* data showing that pharmacological targeting of VGSCs with phenytoin significantly reduces tumour growth, local invasion and metastasis in a mouse model of BCa. Indeed, given that the membrane potential (V_m_) of cancer cells is relatively depolarized [23], and that phenytoin displays robust use-dependent and tonic channel block at depolarized voltages [8], our data suggest that phenytoin may be a highly effective VGSC blocker in tumours.

We propose that VGSCs may be useful molecular targets for BCa therapy, and that repurposing FDA-approved, VGSC-targeting AEDs and Class I antiarrhythmic agents, e.g. phenytoin, carbamazepine, flecainide, to cancer may therefore improve outcome. It is possible that phenytoin may be effective in combination with existing conventional therapies, e.g. in the adjuvant setting, which would need to be tested in a randomised controlled clinical trial. In support of this notion, application of VGSC-targeting local anaesthetics during radical prostatectomy associates with substantially reduced recurrence and metastasis [24]. In addition, the FDA-approved ALS drug, riluzole, which inhibits both metabotropic glutamate receptors and VGSCs, reduces tumour growth [25]. Furthermore, given that VGSCs favour an invasive/metastatic phenotype [9,13-15], it is possible that the adjuvant prescription of AEDs, which cross the blood-brain-barrier, may reduce and/or delay metastasis formation in patients. This would transform the landscape of cancer treatment considerably, with very little added cost, while leading to healthier patients and huge financial savings.

## Additional file

Additional file 1: **Supplementary materials and methods.**

## Abbreviations

AED: Antiepileptic drug
BCa: Breast cancer
CAM: Cell adhesion molecule
LC-SRM-MS: Liquid chromatography-mass spectrometry with single reaction monitoring
MMP9: Matrix metalloproteinase-9
VGSC: Voltage-gated Na^+^ channel

## References

[1] Jemal A, Bray F, Center MM, Ferlay J, Ward E, Forman D (2011). Global cancer statistics. CA Cancer J Clin.

[2] Gupta GP, Massague J (2006). Cancer metastasis: building a framework. Cell.

[3] Brackenbury WJ, Isom LL (2011). Na channel beta subunits: overachievers of the ion channel family. Front Pharmacol.

[4] Brackenbury WJ, Calhoun JD, Chen C, Miyazaki H, Nukina N, Oyama F (2010). Functional reciprocity between Na + channel Nav1.6 and β1 subunits in the coordinated regulation of excitability and neurite outgrowth. Proc Natl Acad Sci U S A.

[5] Mantegazza M, Curia G, Biagini G, Ragsdale DS, Avoli M (2010). Voltage-gated sodium channels as therapeutic targets in epilepsy and other neurological disorders. Lancet Neurol.

[6] Black JA, Waxman SG (2013). Noncanonical roles of voltage-gated sodium channels. Neuron.

[7] Brackenbury WJ (2012). Voltage-gated sodium channels and metastatic disease. Channels (Austin).

[8] Yang M, Kozminski DJ, Wold LA, Modak R, Calhoun JD, Isom LL (2012). Therapeutic potential for phenytoin: targeting Na(v)1.5 sodium channels to reduce migration and invasion in metastatic breast cancer. Breast Cancer Res Treat.

[9] Fraser SP, Diss JK, Chioni AM, Mycielska M, Pan H, Yamaci RF (2005). Voltage-gated sodium channel expression and potentiation of human breast cancer metastasis. Clin Cancer Res.

[10] Roger S, Besson P, Le Guennec JY (2003). Involvement of a novel fast inward sodium current in the invasion capacity of a breast cancer cell line. Biochim Biophys Acta.

[11] Brackenbury WJ, Chioni AM, Diss JK, Djamgoz MB (2007). The neonatal splice variant of Nav1.5 potentiates in vitro metastatic behaviour of MDA-MB-231 human breast cancer cells. Breast Cancer Res Treat.

[12] Brackenbury WJ, Djamgoz MB, Isom LL (2008). An emerging role for voltage-gated Na + channels in cellular migration: regulation of central nervous system development and potentiation of invasive cancers. Neuroscientist.

[13] Brisson L, Driffort V, Benoist L, Poet M, Counillon L, Antelmi E (2013). NaV1.5 Na(+) channels allosterically regulate the NHE-1 exchanger and promote the activity of breast cancer cell invadopodia. J Cell Sci.

[14] House CD, Vaske CJ, Schwartz A, Obias V, Frank B, Luu T (2010). Voltage-gated Na + channel SCN5A is a key regulator of a gene transcriptional network that controls colon cancer invasion. Cancer Res.

[15] Nelson M, Millican-Slater R, Forrest LC, Brackenbury WJ (2014). The sodium channel beta1 subunit mediates outgrowth of neurite-like processes on breast cancer cells and promotes tumour growth and metastasis. Int J Cancer.

[16] Loscher W (2007). The pharmacokinetics of antiepileptic drugs in rats: consequences for maintaining effective drug levels during prolonged drug administration in rat models of epilepsy. Epilepsia.

[17] Borges S, Doppler H, Perez EA, Andorfer CA, Sun Z, Anastasiadis PZ (2013). Pharmacologic reversion of epigenetic silencing of the PRKD1 promoter blocks breast tumor cell invasion and metastasis. Breast Cancer Res.

[18] Gillet L, Roger S, Besson P, Lecaille F, Gore J, Bougnoux P (2009). Voltage-gated sodium channel activity promotes cysteine cathepsin-dependent invasiveness and colony growth of human cancer cells. J Biol Chem.

[19] Pappalardo LW, Samad OA, Black JA, Waxman SG (2014). Voltage-gated sodium channel Nav 1.5 contributes to astrogliosis in an in vitro model of glial injury via reverse Na+ /Ca2+ exchange. Glia.

[20] Abdul M, Hoosein N (2001). Inhibition by anticonvulsants of prostate-specific antigen and interleukin-6 secretion by human prostate cancer cells. Anticancer Res.

[21] Fraser SP, Salvador V, Manning EA, Mizal J, Altun S, Raza M (2003). Contribution of functional voltage-gated Na^+^ channel expression to cell behaviors involved in the metastatic cascade in rat prostate cancer: I. lateral motility. J Cell Physiol.

[22] Yildirim S, Altun S, Gumushan H, Patel A, Djamgoz MB (2012). Voltage-gated sodium channel activity promotes prostate cancer metastasis in vivo. Cancer Lett.

[23] Yang M, Brackenbury WJ (2013). Membrane potential and cancer progression. Front Physiol.

[24] Biki B, Mascha E, Moriarty DC, Fitzpatrick JM, Sessler DI, Buggy DJ (2008). Anesthetic technique for radical prostatectomy surgery affects cancer recurrence: a retrospective analysis. Anesthesiology.

[25] Speyer CL, Smith JS, Banda M, Devries JA, Mekani T, Gorski DH (2012). Metabotropic glutamate receptor-1: a potential therapeutic target for the treatment of breast cancer. Breast Cancer Res Treat.

