# Supplementary material for: The sodium channel-blocking antiepileptic drug phenytoin inhibits breast tumour growth and metastasis
Source: Mol Cancer. 2015 Jan 27;14(1):13. doi: 10.1186/s12943-014-0277-x (PMC4320839; doi:10.1186/s12943-014-0277-x)
Supplement: Supplementary file 1 — Supplementary materials and methods. [file 12943_2014_277_MOESM1_ESM.pdf]

Supplementary materials and methods

Cell culture

MDA-MB-231 cells stably expressing enhanced GFP and firefly luciferase [1] were grown in DMEM supplemented with 5 % FBS and 4 mM L-glutamine [2]. Molecular identity was confirmed by short tandem repeat analysis according to ATCC standards. Cells were confirmed as mycoplasma-free using the DAPI method.

Orthotopic breast tumour model

All animal procedures were performed after approval by the University of York Ethical Review Process and under authority of a UK Home Office Project Licence. Six week-old female *Rag2*$^{-/-}$ *Il2rg*$^{-/-}$ mice were obtained from the Yorkshire Cancer Research Unit, University of York, (3-5 per specific pathogen free cage). 1x10^6 MDA-MB-231 cells suspended in Matrigel (20 % v/v in saline) were injected into the left inguinal mammary fat pad of each animal whilst under isoflurane anaesthesia. A total of 29 mice were used across 8 cages. Individual cages served as independent replicated experiments: each cage contained both control and drug-treated animals (identified by ear notches), in order to reduce cage-to-cage variability and bias. Starting 7 days following implantation of tumour cells, mice within each cage were randomised and given daily intraperitoneal injection of phenytoin sodium (60 mg/kg; Sigma) or vehicle (PBS, pH 10.4) for 4 weeks. Tumour growth was monitored weekly by bioluminescence imaging [1]. Animal weight and the length and width of each tumour (in mm) were measured every 2-4 days. Tumour volume was calculated as 0.5 x (length x width^2). Mice were euthanized 4 weeks following implantation of tumour cells and metastatic bioluminescence was measured [1]. Tumours and organ sites of metastasis were fixed in 4 % paraformaldehyde and frozen [3].

Measurement of plasma phenytoin level

In a subset (n=12) of animals, at 16 h following last phenytoin injection, blood (~500 µl) was taken by cardiac puncture under terminal anaesthesia with medetomidine-ketamine. Whole blood was transferred to EDTA-coated Microtainer tubes (BD Bioscience). Plasma was separated from erythrocytes and buffy coat by centrifugation at 2000 g for 5 min and then stored at -80 ˚C. Phenytoin concentration was measured by liquid chromatography-mass spectrometry with single reaction monitoring (LC-SRM-MS) using metaxalone as an internal standard [4]. Standards for calibration and quality control were generated by spiking varying amounts of phenytoin into blank plasma: triplicate calibration samples at seven concentrations spanning 0.5-200 µg/ml; five quality-control replicates at 1, 3, 15, and 75 µg/ml. Phenytoin extraction was performed in triplicate from 10 µl plasma aliquots with addition of 90 µl acetonitrile [5] containing 0.5 µg/ml metaxalone. After centrifugation at 9000 g for 10 min, the top 50 ml was diluted 1:1 with aqueous 2 % formic acid for LC-SRM-MS analysis using a Dionex UltiMate 3000 HPLC and Applied Biosystems API 3000 triple-quadrupole mass spectrometer. HPLC separation was performed using a Waters XTerra MS C18 column (3.5 mm, 3.0 x 50 mm) with isocratic elution at 1 ml/min with aqueous 35 % acetonitrile containing 1 % formic acid. Positive-ion electrospray ionisation was performed using a TurboIonSpray source. The following precursor and product ions were monitored: phenytoin precursor 262.3 *m/z*, product 182.0 *m/z*; metaxalone precursor 222.2 *m/z*, product 161.1 *m/z*. Data analysis was performed with Analyst software (Applied Biosystems, version 1.4.2). Phenytoin was by quantified from the peak areas of the phenytoin and metaxalone product ions in extracted-ion chromatograms using a standard curve fitted from the calibration samples using a weighting of $1/x^2$.

Immunohistochemistry

H&E stained sections were scanned at 20X using a Zeiss AxioScan.Z1 slide scanner. The following primary antibodies were used for immunohistochemistry [3]: rabbit anti-Na_v1.5 (1:100; Alomone); rabbit anti-Na_v1.7 (1:1000; Abcam); rabbit anti-MMP9 (1:5000; Abcam); rabbit anti-Ki67 (1:5000; Abcam); rabbit anti-activated caspase-3 (1:200; R&D Systems); rabbit anti-CD31 (Santa Cruz Biotechnology); mouse anti-GFP (1:1000; NeuroMab).

Secondary antibodies were Alexa-568-conjugated goat anti mouse/rabbit (1:500; Invitrogen). Tyramide signal amplification was used for matrix metalloproteinase-9 (MMP9) [6]. Samples were mounted in Prolong Gold with DAPI (Invitrogen). Samples were viewed using 20X objectives on a Nikon Eclipse TE200 microscope, or Zeiss Axio Observer.Z1 microscope with LSM 710 confocal laser scanner. Images were exported into ImageJ for processing. Confocal Z-series projections were flattened using the maximum signal. Brightness/contrast was adjusted using the ImageJ “Auto” function. Density of $MMP9^+$, $Ki67^+$ or activated caspase-3^+ cells, tumour vascularity and metastasis to liver/lungs/spleen were measured blinded to treatment [1].

Data analysis

Data are mean and SEM unless stated otherwise. Statistical analysis was performed using GraphPad Prism 6. Pairwise statistical significance was determined with t-tests or Mann-Whitney tests, as appropriate. Metastatic bioluminescence data were log-transformed and analysed by two-way ANOVA. Results were considered significant at $P<0.05$.

References

1. Nelson M, Millican-Slater R, Forrest LC, Brackenbury WJ: **The sodium channel beta1 subunit mediates outgrowth of neurite-like processes on breast cancer cells and promotes tumour growth and metastasis.** *Int J Cancer* 2014, **135:**2338-2351.
2. Yang M, Kozminski DJ, Wold LA, Modak R, Calhoun JD, Isom LL, Brackenbury WJ: **Therapeutic potential for phenytoin: targeting Na(v)1.5 sodium channels to reduce migration and invasion in metastatic breast cancer.** *Breast Cancer Res Treat* 2012, **134:**603-615.
3. Brackenbury WJ, Davis TH, Chen C, Slat EA, Detrow MJ, Dickendesher TL, Ranscht B, Isom LL: **Voltage-gated Na^+ channel β1 subunit-mediated neurite outgrowth requires fyn kinase and contributes to central nervous system development in vivo.** *J Neurosci* 2008, **28:**3246-3256.
4. Roy SMN, Yetal SM, Vaidya VV, Joshi SS: **Determination and Quantification of Phenytoin in Human Plasma by Liquid Chromatography with Electrospray Ionization Tandem Mass Spectrometry.** *E-Journal of Chemistry* 2008, **5:**169-176.
5. Kim KB, Seo KA, Kim SE, Bae SK, Kim DH, Shin JG: **Simple and accurate quantitative analysis of ten antiepileptic drugs in human plasma by liquid chromatography/tandem mass spectrometry.** *J Pharm Biomed Anal* 2011, **56:**771-777.
6. Brackenbury WJ, Yuan Y, O'Malley HA, Parent JM, Isom LL: **Abnormal neuronal patterning occurs during early postnatal brain development of Scn1b-null mice and precedes hyperexcitability.** *Proc Natl Acad Sci U S A* 2013, **110:**1089-1094.
